# Injection of Anabolic Steroids in Men Who Had Sex with Men in Madrid and Barcelona: Prevalence Correlates and Role as a Risk Factor for Transmitted Infections

**DOI:** 10.3390/ijerph18168289

**Published:** 2021-08-05

**Authors:** Juan-Miguel Guerras, Juan Hoyos, Luis de la Fuente, Francisca Román, Oskar Ayerdi, Jorge-Néstor García-Pérez, Patricia García de Olalla, María-José Belza, the Methysos Project Group

**Affiliations:** 1 Centro Nacional de Epidemiología, Instituto de Salud Carlos III, 28029 Madrid, Spain; jguerras@isciii.es (J.-M.G.); lfuente@isciii.es (L.d.l.F.); 2 CIBER Epidemiología y Salud Pública (CIBERESP), 28029 Madrid, Spain; polalla@aspb.cat (P.G.d.O.); mbelza@isciii.es (M.-J.B.); 3 Departamento de Salud Pública y Materno-Infantil, Universidad Complutense de Madrid, 28040 Madrid, Spain; 4 Servicio de Epidemiología, Agència de Salut Pública de Barcelona, 08023 Barcelona, Spain; francisca.roman.urr@gmail.com; 5 Centro Sanitario Sandoval, Instituto de Investigación Sanitaria San Carlos, Hospital Clínico San Carlos, 28010 Madrid, Spain; oskarayerdi@hotmail.com; 6 Unidad de ITS de Vall d’Hebron-Drassanes, Hospital Vall d’Hebron, 08001 Barcelona, Spain; g.perez@vhebron.net; 7 Escuela Nacional de Sanidad, Instituto de Salud Carlos III, 28029 Madrid, Spain

**Keywords:** steroids, injection, MSM, risk behaviour

## Abstract

This study describes the prevalence of anabolic-androgenic steroid (AAS) injection, their main correlates, and the prevalence of specific AAS injection risk behaviours among men who have sex with men (MSM), an area insufficiently addressed in scientific research. Participants were HIV-negative MSM attending four HIV/STI diagnosis services: two clinics and two community programmes in Madrid and Barcelona. Participants answered an online self-administered questionnaire. Crude and adjusted lifetime prevalence and prevalence ratios (PRs) were calculated by different factors and using Poisson regression models with robust variance. Of the 3510 participants, 6.1% (95% CI: 5.3–6.9) had injected AAS before and 3.5% (95% CI: 2.9–4.2) had done so in the last 12 months. In the multivariate analysis, AAS injection was independently associated with being over 40 years old (aPR = 3.6; 95% CI: 2.0–6.5) and being born in Latin America (aPR = 2.5; 95% CI:1.9–3.4), and was less strongly associated (aPRs of around two) with having been recruited into STI clinics, having ever been paid for sex before, injected drugs, used drugs for sex, having been diagnosed with an STI before, and having been diagnosed with HIV at the recruitment consultation. Only three participants, 1.4%, of those who had injected AAS before had shared AAS or equipment for preparation or injecting before. Conclusions: In contrast to drugs, AAS injecting behaviours do not play a relevant, direct role in the transmission of blood-borne infections among MSM. However, AAS injectors have a higher prevalence of sexual risk behaviours. These findings should be confirmed using new studies that employ other sampling procedures.

## 1. Introduction

Anabolic-androgenic steroids (AAS) are a group of natural or synthetic substances used for the treatment of different health problems. However, they are also used illicitly or non-medically, often for the purposes of building muscle and influencing appearance and performance in bodybuilding [1,2]. In fact, different typologies of users have been described based on their concrete motivations or goals for using AAS [3]. AAS use seems to have few serious medical consequences in the short-term, however, it has been associated with several debilitating physical and psychological symptoms and increased mortality in the long-term [4].

A meta-analysis published in 2014 concerning the global epidemiology of AAS showed that the lifetime prevalence of AAS use was highest among recreational sports enthusiasts and athletes, followed by prisoners and arrestees, drug users, high-school students, and finally non-athletes. Intramuscular injection is the most common route of administration [4]. For this reason, the injection of AAS has become a research topic in the last decade of the past century but from a perspective primarily concerned with the potential effects on the spread of HIV and hepatitis B or C in the framework of the drug injection epidemic, especially in the UK [5,6,7,8,9,10]. Most of these studies were carried out with samples recruited in gyms or in needle and syringe exchange programmes. Studies in samples from gyms revealed that most people who had used AAS had injected them, although they had rarely shared preparation or injection equipment, and revealed that AAS injection was more common among men who had sex with other men (MSM) and who had also injected other drugs [8]. A review published in 2016 [11] concerning the prevalence of infectious diseases and risk behaviours among AAS users showed that 3.5% of a group of 3100 AAS users from 20 studies had shared needle/syringes and 13.3% had shared vials or multi-dose containers. These prevalences are much lower than those found among people who injected drugs [12,13,14,15]. Some recent studies carried out among people who injected image and performance enhancing substances (mostly AAS) have raised concerns about these infections in this population [16,17,18]. Conversely, the review mentioned above [11] concluded that AAS injectors showed higher levels of sexual risk behaviours and HIV, HBV, and HCV infection than non-users.

The use and injection of AAS among MSM has attracted much less attention. In fact, there is no reference to this sub-population in the aforementioned meta-analysis [4]. As a recent article [19] emphasized, most of the studies on AAS users have been carried out in samples of mostly heterosexual men, although some of the pioneering studies were focused on MSM, recruited in gyms [7,8]. However, in the last decade, three studies specifically focused on MSM have been published: the EMIS study [20], a recent study in the UK [19], and another study in the USA [21]. EMIS found that 2.6% of MSM had injected AAS before, however, the study did not analyse the correlates. In addition, the study did not provide information on the prevalence of risk behaviours associated with AAS injection as it presented a joint prevalence of having injected drugs or AAS, despite the fact that it is likely the prevalence is different for both types of substances. The EMIS Spanish report [22] has the same limitations. The UK study [19] included a small sample size and low prevalence, and was unable to detect correlates and did not analyse injection risk behaviours. One recent study in the USA among MSM using AAS found greater risky sexual behaviours among MSM AAS-users [21], although the sample size was very modest (N = 150). Only one study [8] has addressed the motivations and health consequences in MSM. The motivations reported for taking steroids in order of frequency were: “to become bigger or stronger”, “to look attractive”, and “for medical reasons”. The most reported side effects were: increased sex drive, testicular atrophy, insomnia, acne/spots, pain at injection sites, and hypertension.

This original study is focused on MSM living in Madrid and Barcelona and without an HIV diagnosis; a priority group from a preventive perspective. The aim of the study was to estimate the lifetime and past year prevalence of AAS injection and to identify their main correlates. It also analysed the prevalence of risky injecting behaviour to determine whether it may play a relevant role in the acquisition of blood-borne infections such as HIV or Hepatitis B or C.

## 2. Materials and Methods

### 2.1. Design, Recruitment, Data Collection Instruments, and Variables

This analysis was based on the first survey of the Methysos Project, which is devoted to analysing the prevalence and characteristics of drug use (including sexualized drug use) among MSM in Spain. The project was approved by the Research Ethics Committee of the Instituto de Salud Carlos III (CEI PI 44_2018_subproyecto1-v2 and CEI PI 44_2018_subproyecto2).

A cross-sectional survey was conducted in four facilities: Sandoval (in Madrid) and Drassanes (in Barcelona), the two most important sexually transmitted infection (STI) clinics in Spain, and the Pink Peace Programme (in Madrid) and the Agència de Salut Pública (in Barcelona), two community programmes for rapid HIV-testing. These STI clinics provide on-demand services and perform traditional testing for all STIs, whereas the community programmes also carry out various kinds of active recruitment including via ads and profiles on dating apps for MSM and only offer rapid testing for HIV, syphilis, and sometimes HCV. The fieldwork of the study was conducted between May 2018 and December 2020. 

The study was limited to men who had sexual intercourse with other men before. We also restricted the study to those without a previous HIV diagnosis because they make up the vast majority of patients at the facilities and are the priority group for prevention of HIV and HCV. All service users without a previous HIV diagnosis of these facilities were offered the opportunity to participate. If they accepted, they were asked to answer a self-administered online questionnaire without personal identifiers on a tablet. This questionnaire included different sections: sociodemographics, history of HIV testing, sexual risk behaviours, both recreational and sexualized drug use, and drug injection for recreational use or in a sexual context. A brief set of questions on injecting AAS was included: “Have you ever injected any anabolic steroids, such as testosterone or other similar substances? When was the last time? How old were you when injected for the first time? Have you ever shared material to prepare the steroids or injection equipment? With how many people have you shared when injecting steroids?” The term “share” included several behaviours because in the questionnaire it was defined as “having injected with a syringe previously used by another person; or, having taken the dissolved substance from a syringe previously used by another person; or, from the container in which another person has previously inserted their syringe”. See appendix for more information on the management of the original variables and categories from the questionnaire to obtain the final variables and categories used in the analysis. The original questionnaire, in Spanish, is available on request from the corresponding author.

In order to detect new HIV infections, the STI clinics performed their usual routine procedures: fourth-generation serologic assays (CMIA in Madrid and CLIA in Barcelona) with confirmation (Western Blot in Madrid and LIA in Barcelona) and quantification of RNA viral load. The two community programmes used point of care rapid tests: in Barcelona, all samples were of capillary blood and Determine TM HIV-1/2^®^ was always used, while in Madrid, 86% were of capillary blood samples and 14% were saliva samples, using Labmen anti-HIV 1/2^®^ (65%), Determine TM HIV-1/2^®^ (18%), and Oraquick HIV^®^ (17%). From all of the participants, 84 were considered as HIV positive in the analysis: 48 from the STI clinics and 36 from the community programmes. Among those 36 who were tested via a rapid test, 20 were also confirmed in the STI clinics, 12 were confirmed by telephone or mail contact, and in 4 cases it was not possible to follow-up for confirmation.

### 2.2. Statistical Analysis

Nearly all of the variables were collected in a more disaggregated form than presented here as some of the original categories were grouped together based on their frequencies and the rationale for the analysis. Descriptive characteristics (n and percentages) of the participants included sociodemographics, sexual risk behaviours, history of HIV testing, and STI diagnosis. Later, we calculated the prevalence of having ever injected before and last-year AAS injection, and this prevalence was stratified by the variables mentioned. To analyse the correlates of having ever injected AAS before, we used Poisson regression models with robust variance. Both crude and adjusted prevalence ratios (cPRs and aPRs) and 95% confidence intervals (95% CI) were calculated [23,24]. After collapsing the number of categories for some variables due to the limited number of participants who had ever injected before, variables with a significance level of <0.25 in the bivariate analysis were introduced into the multivariate model. In order to select the final model, we used the Akaike Information Criteria to perform model comparisons. Finally, we performed a stratified analysis by city, age of first injection, most recent injection, and occurrence of having ever shared AAS or injection equipment before and the number of people shared with. Comparisons of the distributions of these variables by city were assessed using Pearson’s χ2 and Fisher’s exact tests at *p* <0.05. 

In order to explore to what extent the COVID-19 pandemic and its related policies of social control could influence our findings, we performed a sensitivity analysis and stratified our analysis by those recruited before the general population confinement in Spain on 15 March 2020 versus those recruited after the confinement period.

## 3. Results

The study included 3510 participants: 2409 in Madrid (1225 in the STI clinic and 1184 in the community programme) and 1101 in Barcelona (418 in the STI clinic and 683 in the community programme). In terms of sociodemographic characteristics (Table 1), 73.8% of the participants were under 40 years of age, 38.0% were born abroad, 75.9% lived in the cities of Madrid or Barcelona, 59.6% had university-level studies, 60.0% had a comfortable economic situation, and 40.0% had lived alone during the last 12 months. In terms of sexual characteristics and risk behaviour (Table 2); 62.5% had only ever had sex with men; 17.5% had their first sexual relationship with a man before 16 years of age; 61.5% exercised their sexual life with men openly; 64.4% had met most of their partners through websites or dating apps; 5.3% had never been penetrated before; 25.6% had been penetrated by more than 50 men in their lives; 21.5% had been paid for sex before; 17.2% had paid for sex before; and 2.1% had injected drugs before. In relation to HIV and STI testing, 2.4% were diagnosed with an HIV infection during the consultation of the study; 48.8% had been tested for HIV in the last 6 months; 6.4% had never been tested for HIV before; and 71.9% had been diagnosed with an STI at some point during their lives. 

Of all of the participants, 214 (6.1%; 95% CI: 5.3–6.9) had injected AAS before and 124 (3.5%; 95% CI: 2.9–4.2) had done so in the past 12 months. In the bivariate analysis, having ever injected AAS before was associated (cPRs with 95% confidence intervals that did not include the null value) with several different variables (Table 1): having been recruited in STI clinics (cPR = 1.5; 95% CI: 1.2–1.9), being over 25 years old (cPR = 3.0; 95% CI: 1.7–5.2), being born in Latin America (cPR = 2.9; 95% CI: 2.2–3.8), and having secondary or a lower level of education (cPR = 1.7; 95% CI: 1.0–2.6). It was also associated (Table 2) with having ever had sex with women before (cPR = 1.6; 95% CI: 1.3–2.0), having found the largest number of partners at discos/saunas/private parties (cPR = 1.5; 95% CI: 1.1–2.0), having been penetrated by more than 50 men during one’s lifetime (cPR = 2.5; 95% CI: 1.2–5.3) and by more than five men in the past 12 months (cPR = 1.6; 95% CI: 1.1–2.3), having ever been paid for sex before (cPR = 2.8; 95% CI: 2.2–3.6), having ever paid for sex (cPR = 1.8; 95% CI: 1.4–2.4), having ever injected drugs (cPR = 3.2; 95% CI: 1.9–5.5), having ever used drugs for sex (cPR = 2.7; 95% CI: 2.0–3.7), having ever been diagnosed with an STI (cPR = 2.4; 95% CI: 1.7–3.5), having the most recent HIV test < 6 months (cPR = 9.2; 95% CI: 2.3–37.0), and having been diagnosed with HIV at the recruitment consultation (cPR = 2.7; 95% CI: 1.5–4.6). 

In the multivariate analysis (Table 3), having ever injected AAS before was independently associated with being between 25–39 years of age (aPR = 2.8; 95% CI: 1.6–4.9) or over 40 years of age (aPR = 3.6; 95% CI: 2.0–6.5) and with being born in Latin America (aPR = 2.5; 95% CI: 1.9–3.4). It was also associated, though less strongly (aPRs of around two), with having been recruited into STI clinics, having been paid for sex, having injected drugs, having used drugs for sex, having been diagnosed with an STI, having been diagnosed with HIV during the recruitment consultation, and, at an even weaker association, having had sex with women before (aPR = 1.4; 95% CI: 1.1–1.8). 

Only 7.5% of participants had injected steroids for the first time before the age of 20, while 21.5% did so at the age of 35 or older; 58.0% had injected steroids within the last year and 17.8% had last injected more than 5 years ago. Only three participants, 1.4%, of those who had ever injected AAS before had shared the substance or injection equipment before. Of these three, two had also injected illegal drugs and one participant had shared when injecting both AAS and drugs. Of the three, one had shared only with one person and the other two had shared with less than five people. There were no significant differences in the patterns of AAS injections between Madrid and Barcelona except that in Barcelona, the proportion of AAS injectors who had ever injected drugs before was much higher (3.9% vs. 13.1%) (Table 4). 

Only 18% of the participants were recruited after the general population confinement, all of them in two of the four programs. There were no significant differences between those recruited before and after the confinement for the main variables of the study: lifetime and last-year AAS injecting prevalence, time since last injection, and risky injecting behaviours. In relation to the correlates with having ever injected AAS before, there were significant differences in two variables but only in the program in Madrid, not in Barcelona’s program. Those recruited after the confinement showed a higher percentage of having a “tight/very difficult” economic situation and of being “unemployed”. When we restricted the multivariate analysis to those enrolled before the pandemic, the correlates were the same as in the total sample and the strength of the prevalence ratios was very similar.

## 4. Discussion

### 4.1. Main Results

To our knowledge, this is the first study to analyse not only the prevalence of AAS injection among MSM but also its correlates, as well as the sharing of AAS or injecting equipment. Some 6.1% of participants had injected these substances before and more than half had done so within the last 12 months. This behaviour did not begin early in life as two out of five participants began at age 30 or older and one in five at age 35 or older. Participants born in Latin America had an adjusted prevalence of 2.5 times higher than those born in Spain and those who had sex with women before had an aPR of 40% higher than those who only had sex with men before. AAS injecting behaviours did not appear to be a relevant direct risk factor for the spread of blood-borne infections as less than one per hundred participants had shared the injection substances or equipment. However, injecting AAS was independently associated with having been diagnosed with HIV at the study recruitment consultation. In addition, having injected AAS before was also independently associated with other behaviours that are widely recognised in the scientific literature as risk factors for the transmission of HIV and other infections, including having been paid for sex before, having injected drugs, having used drugs for sex, and having been diagnosed with STIs.

### 4.2. Discussion of the Results and Comparisons with Other Studies

It is difficult to compare the prevalence of AAS injection found in the current study with most of the studies on steroid injections. Our study was focused on MSM while most of the studies that have included MSM used intentional samples not restricted to MSM. Most of them recruited men in gyms or in services oriented to people who inject drugs. In addition, some of the latter were restricted to AAS injectors only. The prevalence of having injected steroids before found in our study (6.2%) is almost double that found by the EMIS Study in 2017 in the Spanish sample recruited primarily through gay contact apps/websites (3.7%) [22]. In contrast to our study, EMIS also included HIV-positive MSM who probably have a higher prevalence of steroid injection, as our study also shows. Therefore, the difference between the two studies could be because MSM samples that demand testing for HIV or other STIs tend to select people with higher risk behaviours. The twelve-month prevalence of the current study (3.5%) is similar to that found in MSM in the UK LGBT study [19]. Bearing in mind that this study also recruited HIV-positive MSM, it asked not only about steroids but also about other image and performance enhancing substances (IPES), and it was not restricted by the injection route. This study suggests that the prevalence in MSM living in these Spanish cities is substantially higher than in the UK. 

In this study, the age of first injection of AAS (29 years) was very similar to the age of the first injection of drugs in this population [25], although both are clearly higher than the age of first drug injection in the general population in Spain [26]. The two studies on steroid use in MSM mentioned above did not provide this information [19,22]. The age of first injection and the cross-sectional design of this study explains the prevalence that is associated with the increasing age.

Having been born in Latin America was the second strongest correlate in our study, after adjusting for other factors. Half of this group had first injected before coming to Spain, thus it could be that that this behaviour is linked to cultural differences and is not necessarily related to the impact of Spanish culture. One study in the USA found that Black and especially Hispanic sexual minority adolescents used more AAS than did White minorities [27].

This study provides clear evidence that AAS injection behaviours do not seem to play any relevant, direct role in the transmission of infections such as HIV or hepatitis B or C because only 1.4% of MSM who had injected AAS before, less than one in a thousand of the global MSM sample, had shared AAS or injecting equipment before. It should be noted that in our study, the term “sharing” was defined in a detailed and broad manner (as explained in the methods section) and was not limited to syringe sharing. The review on AAS injection cited [11]—which mostly concerns non-MSM populations—emphasizes that syringes are shared less than the substance, the containers, and/or other paraphernalia. However, an intermediate analysis of our study showed that more than one in three participants had shared when injecting drugs before [25]. Therefore, this data confirms our hypothesis that jointly analysing risk behaviours related to drug and AAS injection is not recommended, as they are radically different. The lack of differentiation of the two substances and the failure to stratify results in analyses has led to inaccurate prevalences that in no way reflect what happens with steroid injection but rather with drug injection. This has occurred both in studies that have sampled MSM [20,22] and in those that have sampled steroid or IPES injectors [16,17,18].

Although AAS injection behaviours did not pose a direct risk for HIV or HCV infections, this study showed that being an AAS injector was independently associated with the incidence of HIV. This association can be explained by the fact that being an AAS injector was also independently associated with other behaviours that are widely recognized as risk factors for HIV, HCV, or HBV: being paid for sex, injecting drugs, or using drugs for sex. Therefore, knowledge about AAS injection characteristics among MSM should alert health workers to the need to inquire about these other associated risk behaviours.

### 4.3. Strengths and Limitations

This study enrolled a significant sample of MSM from two programmes with very different client recruitment characteristics that were selected in each city in order to increase the sample’s heterogeneity and representativeness. A self-administered questionnaire was used in order to reduce the reporting bias of socially sanctioned behaviours.

However, like almost all studies in this area, we used a convenience sample: in this case, MSM demanding HIV testing. In general, convenience samples tend to include higher-risk MSM than general population surveys [28,29]. Therefore, generalizing results even among HIV-negative MSM should be done with caution. The prevalences found here most likely underestimate the true prevalences of MSM as a whole because they do not include HIV-positive MSM and because being a steroid injector is related to being HIV-positive.

## 5. Conclusions and Implications for Research and Public Health

In contrast to drug injection, AAS injection behaviours do not seem to play any relevant, direct role in the transmission of blood-borne infections in MSM. Although more than one in 20 MSM had injected steroids before, less than one per thousand had ever shared the AAS or injecting equipment before. However, MSM who inject these substances are at increased risk of HIV and other STS infections, most likely because they also have a higher prevalence of sexual risk behaviours. Thus, interventions among these individuals should focus primarily on sexual risk behaviours and not on injecting behaviours, unless they have injected drugs.

Therefore, more studies are needed in order to confirm these novel findings. Considering the difficulty of obtaining representative samples of MSM, these studies should use different types of samples (representative population samples and convenience samples recruited out of the health services), should include HIV positive MSM, should not be restricted to those living in big cities, and should be carried out in different countries and cultural contexts. Moreover, these studies should address other topics not covered in this study, such as the motivations for steroid use and knowledge and perceptions of the effects on health. This would allow for obtaining useful insight to support the design and implementation of specific preventive and harm reduction strategies. Meanwhile, interventions in MSM using AAS must focus primarily on sexual risk behaviours and not on injecting behaviours, unless they have injected drugs using the more general knowledge already available on preventive and harm reduction strategies in these two types of behaviours.

## Figures and Tables

**Table 1:** Sample characteristics, prevalence of having ever injected steroids before, and bivariate analysis of factors associated among MSM * in Madrid and Barcelona (I).

	Sample Characteristics		Prevalence of Having Ever Injected Steroids before	Crude Prevalence Ratio	(95% CI **)
	N = 3510		N = 214 (6.1%)		
	N	%	%		
	**Recruitment**				
**City of testing**					
Madrid	2409	68.6	6.4	1.1	0.9–1.5
Barcelona	1101	31.4	5.5	1.0	
**Kind of testing programme**					
Community programme	1867	53.2	4.9	1.0	
STI diagnostic clinics	1643	46.8	7.4	**1.5**	1.2–1.9
	**Sociodemographics**				
**Age (years)**					
<25	576	16.4	2.3	1.0	
25–39	2015	57.4	6.7	**3.0**	1.7–5.2
≥40	919	26.2	7.1	**3.1**	1.8–5.6
**Country of birth**					
Spain	2177	62.0	4.0	1.0	
Latin America	960	27.4	11.5	**2.9**	2.2–3.8
Others	373	10.6	4.8	1.2	0.8–2.0
**Size of city of residence (last 12 months)**					
>1 million	2649	75.9	5.9	1.0	
100.000–1 million	444	12.7	6.3	1.1	0.8–1.6
≤100.000	398	11.4	7.0	1.2	0.8–1.7
**Level of education**					
Upper secondary	243	6.9	9.1	**1.7**	1.0–2.6
Post-secondary	1169	33.4	6.5	1.2	0.9–1.5
University	2085	59.6	5.5	1.0	
Employment status (last 12 months) ***					
Employed	1810	74.4	6.7	1.0	
Unemployed	190	7.8	10.5	1.6	0.9–2.5
Others	433	17.8	2.5	0.4	0.3–0.6
**Economic situation (last 12 months)**					
Comfortable/It is OK	2101	60.0	6.1	1.0	
Tight	1097	31.3	5.3	0.9	0.7–1.1
Difficult/Very difficult	302	8.6	8.6	1.4	0.9–2.1
Cohabitation (last 12 months) ***					
With some people	1462	60.0	5.7	1.0	
Alone	974	40.0	7.1	1.2	0.9–1.6

* MSM: men who have sex with men. ** 95% CI: 95% confidence interval. *** These questions were not included in Barcelona assessments. Bold number: the bold helps to quickly identify the name of the variables and the significant prevalence ratio but they can be eliminated.

**Table 2:** Sample characteristics, prevalence of having ever injected steroids before, and bivariate analysis of factors associated among MSM * in Madrid and Barcelona (II).

	Sample Characteristics		Prevalence of Having Ever Injected Steroids before	Crude Prevalence Ratio	(95% CI **)
	N = 3510		N = 214 (6.1%)		
	N	%	%		
	**Sexual and risk behaviour**				
**Gender of sex partners (ever)**					
Only men	2194	62.5	5.0	1.0	
Men and women	1316	37.5	8.0	**1.6**	1.3–2.0
**Age at first sexual intercourse with another man (years)**					
≤15	614	17.5	6.7	1.1	0.7–1.7
16–20	1902	54.2	6.3	1.0	0.7–1.5
21–24	566	16.1	4.9	0.8	0.5–1.3
≥25	424	12.1	6.1	1.0	
**Exercised sex life with men…**					
Not Openly	1344	38.5	5.7	1.0	
Openly	2149	61.5	6.3	1.1	0.9–1.4
**Place where the largest number of partners were found**					
Discos/clubs/bars	516	15.3	8.1		
Saunas	327	9.7	7.0		
Apps/webs	2177	64.4	5.3		
Cruising places	113	3.3	5.3		
Private parties	115	3.4	10.4		
Others/no search	131	3.9	4.6		
**Place where the largest number of partners were found**					
Discos/bars/saunas/private parties	958	28.4	8.0	**1.5**	1.1–2.0
Others	2421	71.7	5.3	1.0	
**Number of men who have penetrated (ever)**					
None	187	5.3	3.7	1.0	
≤50	2426	69.1	5.1	1.4	0.7–2.9
>50	897	25.6	9.3	**2.5**	1.2–5.3
**Number of men who have penetrated you (last 12 months)**					
None	716	20.4	4.9	1.0	
≤5	1630	46.5	5.5	1.1	0.8–1.6
>5	1160	33.1	7.7	**1.6**	1.1–2.3
**Having ever been paid for sex**					
No	2754	78.5	4.4	1.0	
Yes	755	21.5	12.3	**2.8**	2.2–3.6
**Having ever paid for sex**					
No	2906	82.8	5.3	1.0	
Yes	604	17.2	9.8	**1.8**	1.4–2.4
**Having ever injected drugs**					
No	3431	97.9	5.8	1.0	
Yes	75	2.1	18.7	**3.2**	1.9–5.5
**Having ever used drugs for sex**					
No	1558	44.5	3.2	1.0	
Yes	1942	55.5	8.5	**2.7**	2.0–3.7
	**History of HIV and other STI testing**				
**STI diagnosis (ever)**					
No	978	28.1	3.1	1.0	
Yes	2498	71.9	7.4	**2.4**	1.7–3.5
**Time since last HIV test**					
≤6 months	1710	48.8	8.2	**9.2**	2.3–37.0
>6 months	1570	44.8	4.6	**5.1**	1.3–20.9
Never tested before	224	6.4	0.9	1.0	
**HIV diagnosis during the recruitment consultation**					
No	3374	97.6	5.8	1.0	
Yes	84	2.4	14.5	**2.7**	1.5–4.6

* MSM: men who have sex with men. ** 95% CI: 95% confidence interval. Bold number: the bold helps to quickly identify the name of the variables and the significant prevalence ratio but they can be eliminated.

**Table 3:** Multivariate regression analysis of factors associated with having ever injected steroids before among MSM * in Madrid and Barcelona.

	Adjusted Prevalence Ratio	(95% CI **)
**Kind of testing programme**		
Community programme	1.0	
STI diagnostic clinics	**1.8**	1.4–2.3
**Age (years)**		
<25	1.0	
25–39	**2.8**	1.6–4.9
≥40	**3.6**	2.0–6.5
**Country of birth**		
Spain	1.0	
Latin America	**2.5**	1.9–3.4
Others	1.2	0.7–1.9
**Gender of sex partners (ever)**		
Only men	1.0	
Men and women	**1.4**	1.1–1.8
**Having ever been paid for sex**		
No	1.0	
Yes	**1.9**	1.5–2.6
**Having ever injected drugs**		
No	1.0	
Yes	**1.9**	1.1–3.1
**Having ever used drugs for sex**		
No	1.0	
Yes	**1.9**	1.4–2.6
**STI diagnosis (ever)**		
No	1.0	
Yes	**1.9**	1.3–2.7
**HIV diagnosis during the recruitment consultation**		
No	1.0	
Yes	**2.0**	1.2–3.2

* MSM: men who have sex with men. ** 95% CI: 95% confidence interval. Bold number: the bold helps to quickly identify the name of the variables and the significant prevalence ratio but they can be eliminated.

**Table 4:** Characteristics of having ever injected steroids before among MSM * in Madrid and Barcelona.

	Madrid		Barcelona		Total		*p*-Value **
	N = 153 (6.4%)		N = 61 (5.5%)		N = 214 (6.1%)		
	N	%	N	%	N	%	
**Age of first injection**							0.332
<20	13	8.5	3	4.9	16	7.5	
20–24	33	21.6	9	14.8	42	19.6	
25–29	50	32.7	18	29.5	68	31.8	
30–34	29	19.0	13	21.3	42	19.6	
≥35	28	18.3	18	29.5	46	21.5	
**Last time injected**							0.287
Last month	28	18.3	16	26.2	44	20.6	
1–6 months	34	22.2	17	27.9	51	23.8	
7–12 months	21	13.7	8	13.1	29	13.6	
1–5 years	38	24.8	14	23.0	52	24.3	
>5 years	32	20.9	6	9.8	38	17.8	
**Having ever shared steroids or injection equipment**							0.14
Yes	1	0.7	2	3.3	3	1.4	
No	152	99.3	59	96.7	211	98.6	
**With how many people**							0.386
1	0	0.0	1	50.0	1	33.3	
2–5	1	100.0	1	50.0	2	66.7	
**Having ever injected drugs**							**0.014**
Yes	6	3.9	8	13.1	14	6.5	
No	147	96.1	53	86.9	200	93.5	

* MSM: Men who have sex with men. ** Fisher’s exact tests. Bold number: the bold helps to quickly identify the name of the variables and the significant prevalence ratio but they can be eliminated.

## Data Availability

The datasets used and/or analysed during the current study are available from the corresponding author on reasonable request.
